# Aggregation-Inhibiting scFv-Based Therapies Protect Mice against AAV1/2-Induced A53T-α-Synuclein Overexpression

**DOI:** 10.3390/biom13081203

**Published:** 2023-07-31

**Authors:** Benjamin W. Schlichtmann, Bharathi N. Palanisamy, Emir Malovic, Susheel K. Nethi, Piyush Padhi, Monica Hepker, Joseph Wurtz, Manohar John, Bhupal Ban, Vellareddy Anantharam, Anumantha G. Kanthasamy, Balaji Narasimhan, Surya K. Mallapragada

**Affiliations:** 1 Department of Chemical and Biological Engineering, Iowa State University, Ames, IA 50011, USA; bwschlichtmann@gmail.com (B.W.S.); snethi@iastate.edu (S.K.N.); 2 Nanovaccine Institute, Ames, IA 50011, USA; mjohn@pathovacs.com (M.J.); vellareddy.anantharam@uga.edu (V.A.); anumantha.kanthasamy@uga.edu (A.G.K.); 3 Department of Biomedical Sciences, Iowa State University, Ames, IA 50011, USA; nive@iastate.edu (B.N.P.); emalovic@iastate.edu (E.M.); ppadhi@iastate.edu (P.P.); mhepker@iastate.edu (M.H.); jwurtz3@iastate.edu (J.W.); 4 PathoVacs, Incorporated, Ames, IA 50011, USA; 5 Indiana Biosciences Research Institute (IBRI), Indianapolis, IN 46202, USA; banbhupal@gmail.com; 6 PK Biosciences Corporation, Ames, IA 50011, USA; 7 Department of Physiology and Pharmacology, University of Georgia, Athens, GA 30602, USA

**Keywords:** scFv, polyanhydride nanoparticles, AAV, Parkinson’s disease, alpha synuclein

## Abstract

To date, there is no cure for Parkinson’s disease (PD). There is a pressing need for anti-neurodegenerative therapeutics that can slow or halt PD progression by targeting underlying disease mechanisms. Specifically, preventing the build-up of alpha-synuclein (αSyn) and its aggregated and mutated forms is a key therapeutic target. In this study, an adeno-associated viral vector loaded with the A53T gene mutation was used to induce rapid αSyn-associated PD pathogenesis in C57BL/6 mice. We tested the ability of a novel therapeutic, a single chain fragment variable (scFv) antibody with specificity only for pathologic forms of αSyn, to protect against αSyn-induced neurodegeneration, after unilateral viral vector injection in the substantia nigra. Additionally, polyanhydride nanoparticles, which provide sustained release of therapeutics with dose-sparing properties, were used as a delivery platform for the scFv. Through bi-weekly behavioral assessments and across multiple post-mortem immunochemical analyses, we found that the scFv-based therapies allowed the mice to recover motor activity and reduce overall αSyn expression in the substantia nigra. In summary, these novel scFv-based therapies, which are specific exclusively for pathological aggregates of αSyn, show early promise in blocking PD progression in a surrogate mouse PD model.

## 1. Introduction

Parkinson’s disease (PD) is the second most common neurodegenerative disease characterized by loss of motor function. It is a devastating, progressive disease that has no FDA-approved therapeutics that slow or halt disease progression to date. The areas of the brain most affected by PD are the substantia nigra (SN) and the striatum (STR; i.e., nigrostriatal pathway), which contain the cell bodies and projections, respectively, of dopaminergic neurons [1]. The pathogenic hallmark of PD, Lewy bodies are partly composed of the abnormal accumulation of the protein alpha-synuclein (αSyn) [2]. Physiological αSyn has an important role in neuronal homeostasis [3], but phosphorylation of the 129th residue of αSyn (αSyns129) increases the propensity for aggregation of αSyn (i.e., αSyn_agg_) and other proteins which leads to progressive loss of motor function in later stages of PD, making αSyns129 an appropriate toxic marker for PD [4].

Transgenic approaches expressing the A53T mutation of αSyn to induce pathologic morphology indicative of PD pathogenesis are well-studied models for PD research [5,6,7]. Additionally, adeno-associated viral vector (AAV) challenge models for PD have attracted attention in recent years because they have a high affinity for dopaminergic cells [8,9]. The first and second serotypes of AAV are advantageous in combination because they integrate high titer-production and brain tissue-penetrating capabilities [10,11]. One such viral vector that drives the overexpression of mutated αSyn, AAV1/2-A53T, has shown rapid degeneration after injection into the SN, which is also accompanied by the formation of αSyn aggregates and dopaminergic-specific neuronal loss, and has shown PD pathogenesis across multiple animal models [10,11,12,13,14,15]. An important feature of this AAV model is that a significant reduction in dopaminergic neurons is seen only 6 months post-transduction, but the overexpression of α-synuclein alone, even without the neurodegeneration, triggers microglial activation and neuroinflammation, as seen in PD, making it an ideal model to test therapeutics designed to target aggregated α-Syn [12,13].

Most PD therapeutics currently in development either focus on antioxidant therapy or αSyn-targeted therapies [16]. Of these, the latter has attracted more attention recently due to its integral role in PD pathogenesis [17]. αSyn-targeted therapeutics either target different forms of αSyn, ranging from monomeric, mutated, oligomeric, or aggregated forms of αSyn using either antibodies or small molecules, or increase the activity of the autophagy pathway [18]. In this regard, anti-αSyn_agg_ antibodies are a primary point of interest due to their specificity for only pathologic forms of αSyn [19].

Our recent work has identified both polyclonal and monoclonal antibodies and their fragments that have a high affinity specifically for the aggregates of α-Syn [20]. We have also demonstrated in vitro efficacy of the recombinantly produced single-chain fragment variable antibody, namely scFv 3A8 (or simply scFv) [21], in ameliorating and even halting αSyn aggregation [19]. Using the specific pathology induced by the AAV1/2-A53T model described above, this work focuses on evaluating the in vivo efficacy of the scFV therapeutic in this model.

For brain-targeted therapeutics, achieving adequate brain bioavailability is often a significant challenge. In particular, it is unknown if intravenous delivery of scFv can result in brain bioavailability and if this scFv can ameliorate αSyn-induced neurotoxicity in vivo. Nanocarrier-based therapeutic delivery platforms present opportunities to improve therapeutic efficacy while minimizing toxic and systemic side effects [21,22,23] and have received increased attention over the past few years with regard to αSyn-targeted therapies [17]. In particular, polyanhydride nanoparticles (NPs) are promising for use as brain-delivery vehicles because they are highly biocompatible [24,25,26,27], offering precisely tuned release kinetics of encapsulated therapeutics [24,27,28,29,30,31,32,33,34], are efficiently internalized by multiple cell types [35], and provide dose-sparing [36,37,38].

A polyanhydride copolymer based on 10 mol% 1,8,-bis(p-carboxyphenoxy)-3,6-dioxaoctane (CPTEG) and 90 mol% sebacic anhydride (SA) (i.e., 10:90 CPTEG:SA) to encapsulate the scFv therapeutic is of particular interest for treatment of AAV-induced rapid αSyn-associated neurotoxicity because of its ability to provide drug sustained release and high levels of cellular internalization [39]. A recent study evaluated biodistribution of fluorescent dyes administered intravenously (IV) using either a soluble dose, or with dye-encapsulated NPs, where it was found that the overall concentration of dye in the brain persisted for one week [40]. Our hypothesis is that scFv and scFv-encapsulated NPs can enhance therapeutic efficacy of the scFv by ameliorating AAV-induced over-expression of αSyn. The goal of this study is to conduct an initial assessment of the in vivo therapeutic efficacy of these novel scFv 3A8 therapeutics and their NP formulations in C57BL/6 mice using an AAV1/2-A53T-induced rapid αSyn toxicity PD model.

## 2. Materials and Methods

### 2.1. Animals

All animal experiments were conducted in accordance with the protocol approved by the Institutional Animal Care and Use Committee (IACUC) at Iowa State University (Protocols IACUC-19-188 and IACUC-19-269). Male and female C57BL/6 mice were purchased from either Charles River Laboratories (Wilmington, MA, USA) or Jackson Laboratory (Bar Harbor, ME, USA) and kept in a near pathogen-free environment, maintained under standard housing, and controlled temperature and light conditions (21 °C, 12-h dark/light cycle) with free access to food and water.

### 2.2. Polymer Synthesis

A 10:90 CPTEG:SA copolymer was synthesized as described previously [41]. Briefly, the diacids were acetylated at 140 °C for 30 min, and then reacted at 180 °C at 0.2 torr for 30–60 min, dissolved into methylene chloride (Thermo Fisher Scientific, Waltham, MA, USA), precipitated into hexanes (Thermo Fisher Scientific), and dried. The molecular weight of the copolymer was ~15,000 g/mol, as confirmed by proton nuclear magnetic resonance (NMR; MR-400 MHz, Varian, Palo Alto, CA, USA) spectroscopy.

### 2.3. Recombinant scFv Expression

scFv for biodistribution experiments was recombinantly produced in a non-pathogenic *E. coli* expression host strain, TG1 (F’ [traD36 proAB lacIqZ ΔM15] supE thi-1 Δ(lac-proAB) Δ(mcrB-hsdSM)5(rK-mK-; Sigma-Aldrich, St. Louis, MO, USA). The variable regions of heavy chain (HC) and light chain (LC) genes were isolated using standard molecular cloning methods as described in Supplementary Materials). The mouse antibody sequences of heavy and light chains of variable regions were codon-optimized, and the corresponding DNAs were synthesized in an scFv format using a 20 aa (GGGGS) 4 linker in between variable heavy and light chains as described in Supplementary Materials. Specifically, scFv-producing *E. coli* was grown at 37 °C in 5 mL terrific broth (TB) media shaking at 250 rpm overnight. Stocks were stored in 15% glycerol at −80 °C until further use. *E. coli* was then incubated in 250 mL of a 3 L Fernbach flask (cat no. 50202098, Thermo Fisher Scientific) at a starting OD560 of 0.05 at 37 °C, 180 rpm for ~2–3 h, until reaching an OD560 of 0.6. Then, the temperature was reduced to 20 °C, and 0.1 mM isopropyl β-D-1-thiogalactopyranoside (IPTG) was added to induce over-expression and cells were grown overnight for 24 h. Cells were centrifuged at 6000× *g* for 30 min, the supernatant was decanted, and cell pellets were frozen at −80 °C.

To collect recombinant scFv protein, the frozen cell pellets were suspended in a lysis buffer of 20 mM PBS, 100 mM NaCl, and 200 µg/mL chicken egg white lysozyme with EDTA-free protease inhibitors (pH 7.4). The cells were lysed for 3 h in 4 °C in an end-over-end rotator. Then, 20 µg/mL DNAse and 10 mM MgCl_2_ was added to the lysate and mixed thoroughly, followed by end-over-end incubation at room temperature for 20 min. The lysates were centrifuged at 15,000× *g* for 30 min at 4 °C and the soluble fraction protein extract supernatant was collected for purification.

### 2.4. scFv Purification

The soluble fraction supernatant was further purified by a batch method using a polyhistidine-tag resin. Specifically, 5 mL of a nickel–nitrilotriacetic acid resin slurry (50% slurry in 20% ethanol) was added to a 50 mL conical tube. The tube was centrifuged for 2 min at 700× *g*, and supernatant was discarded. The resin was resuspended in two resin-bed volumes of an equilibration buffer, containing 20 mM sodium phosphate with 300 mM sodium chloride (i.e., phosphate-buffered saline, PBS), and 10 mM imidazole (pH 7.4), followed by centrifugation for 2 min at 700× *g*. Then, the protein extract was mixed 1:1 with equilibration buffer and 20 column volumes were added to the resin and mixed for 2 h in an end-over-end rotator. The tube was centrifuged for 2 min at 700× *g* and collected for re-incubation. The resin was washed 5–8 × in 2 column volumes in a wash buffer of PBS with 25 mM imidazole (pH 7.4) followed by centrifugation until the OD280/260 reached baseline levels. Then, the scFv was eluted by incubating the resin for 5 min in 2 column volumes in an elution buffer of PBS in increasing imidazole concentrations, including 2 × each of 250 mM, 350 mM, and 500 mM imidazole, centrifuging between each elution step. Once protein concentrations by OD280/260 again reached baseline, wash buffer was added to rinse the resin 2×, followed by centrifugation. scFv buffer was exchanged for 1× PBS using 10 kDa molecular weight cut-off spin-concentration columns (Cat. No. 88513, Thermo Fisher Scientific) and stored at 4 °C until further use.

### 2.5. scFv Characterization

The presence of purified protein was confirmed by SDS-PAGE and anti-Myc tag dot blot. Specifically, SDS-PAGE and dot blot studies were performed to evaluate the molecular weight and presence of purified scFv, respectively. Briefly, 7 µL scFv sample was added to 63 µL of a solvent containing 4× Laemmli sample buffer (Cat. no. 161-0747, Bio-Rad, Hercules, CA, USA) diluted 1:4 into 1× PBS with 10 mM dithiothreitol (DTT, Thermo Fisher Scientific), which was then heated for 7 min at 100 °C. Approximately 40 µL was loaded into polyacrylamide gels (Cat. no. 4569034, Bio-Rad) for SDS-PAGE studies at 200 V constant voltage at 4 °C for 30 min in 10× Tris/Glycine/SDS buffer (Cat. no. 1610732, Bio-Rad) diluted 10× in dH2O, and visualized via Coomassie blue staining. To evaluate 1-day biodistribution in mice, scFv was first conjugated to VivoTag 800 (cat. no. NEV11107, PerkinElmer, Waltham, MA, USA) using the manufacturer’s protocol. To use scFv for treatment over a 10-week period following AAV injection into mice, endotoxins were first purified from scFv using endotoxin removal resin (Cat. no. 88274, Thermo Fisher Scientific) using the manufacturer’s protocol.

### 2.6. RT-QuIC Assay

αSyn RT-QuIC assay was performed based on previously established protocols with minor modifications to test the activity of antibody on seeding kinetics [42]. In brief, the recombinant αSyn was purified using size exclusion chromatography and anion exchange chromatography and characterized biochemically for purity and activity. The αSyn RT-QuIC was performed using a 96-well clear-bottom plate. The reaction mixture consisted of final concentrations of 40 mM of phosphate buffer (pH 8.0), 170 mM of NaCl, 10 μM of ThT, and 0.1 mg/mL of recombinant αSyn. Before each reaction setup, the recombinant αSyn was filtered through a 100-kDa MWCO filter to retain monomer filtrate. The plates were loaded with six 0.8-mm silica beads (OPS Diagnostics, Lebanon, NJ, USA) per well prior to adding reaction components. Each reaction consisted of 5 μL of test sample in 100 μL of αSyn RT-QuIC reaction mixture per well of a 96-well. The test sample consisted of 2 µL of diluted brain homogenates from Dementia with Lewy bodies (DLB) brain homogenates diluted 4 logs, and 5 µL of scFv at 500 µg/mL (final concentration 25 µg/mL) added separately into 93 µL of the reaction mixture with pre-incubation. Next, the reactions were performed in a CLARIOstar (BMG LABTECH Inc., Cary, NC, USA) plate reader with alternating 1-min shake and rest cycles (double orbital, 400 rpm) at 42 °C. ThT fluorescence readings were recorded at excitation and emission wavelengths of 450 and 480 nm, respectively, every 30 min.

### 2.7. NP Synthesis and Characterization

scFv at 1% w/w was encapsulated into 10:90 CPTEG:SA NPs by an anti-solvent nano-encapsulation method at room temperature, as described previously [41]. Briefly, scFv at 20 mg/mL in 50 µL PBS was added to non-functionalized polymer dissolved in methylene chloride at 20 mg/mL while sonicating at 30% for 30s and immediately precipitated into pentane (Thermo Fisher Scientific) at room temperature at a 1:250 MeCl_2_: pentane ratio. NP morphology and size were determined using field-emission scanning electron microscopy (Quanta 250 FEG, Hillsboro, OR, USA). ImageJ 1.43u software (National Institutes of Health, Bethesda, MD, USA) was utilized to quantify NP size and size distribution. NP release kinetics and encapsulation efficiency were performed in vitro as described previously [43]. Briefly, scFv total protein was determined using a Pierce bicinchoninic acid (BCA) assay (Thermo Fisher Scientific), and the absorbance was recorded using a spectrophotometer at OD570. A standard curve of scFv from 0.5–125 µg/mL was used for quantification.

### 2.8. scFv Biodistribution in C57BL/6 Mice

To evaluate biodistribution of scFv in C57BL/6 mice, 100 µL soluble scFv or NPs was administered IV via lateral tail vein injection at 20 µg scFv (or 2 mg total NP formulation)/kg mouse body weight using a 26-gauge needle. To assist with IV injection, a mouse restraint device was used, together with a heating lamp to dilate the tail vein. Control mice were injected with PBS only. Mice were euthanized and the brain was dissected for further analysis at 2 h and 24 h post-injection. The experiments were performed in quadruplicate. To assess fluorescence levels in mice administered soluble scFv or NPs, brains were dissected and imaged using the IVIS^®^ Spectrum live animal imaging system (Perkin Elmer) at an excitation wavelength of 745 nm and an emission wavelength of 800 nm. To estimate the concentration of scFv in organs, a standard curve of scFv from 0.3–10 µg/mL was obtained on a 96-well plate in triplicate and imaged using the same settings as that for tissues.

### 2.9. Adeno-Associated Vectors (AAV)1/2 Serotype Injection and Treatment Regimen

AAV1/2-A53T-αSyn viral vector was purchased from Vigene (catalog no. GD1001-RV). Deeply anesthetized mice were stereotactically injected unilaterally into the left SN with a microinjector at a rate of 0.5 µL/min with either 1.5 µL of PBS (control) or 1.5 µL of AAV1/2 expressing human mutated A53T-αSyn at a concentration of 5 × 1012 GC/mL. According to the mouse brain atlas of Paxinos and Franklin, the coordinates from Bregma: AP −3.1 mm; ML −1.4 mm; DV −4.4 mm were applied for incision [44]. Six mice were injected with PBS, and 23 mice were injected with AAV1/2-A53T-αSyn. Mice were monitored in the hours and days after surgery to ensure recovery from the incision site. Additionally, mouse weights were monitored weekly. Two weeks after AAV1/2 injection, scFv soluble (n = 8 mice/group) or scFv-encapsulated NP (n = 8 mice/group) treatment was initiated. Specifically, mice were injected IV in the lateral tail vein using a 26-gauge needle 3× weekly at a concentration of 20 µg scFv (or 2 mg total NP formulation for encapsulated dose)/kg mouse body weight until 6 weeks post AAV1/2 injection (i.e., Regimen 1). Then, mice were injected 1× weekly at a concentration of 50 µg scFv (5 mg NPs)/kg mouse body weight for 12 weeks (i.e., Regimen 2). To assist with IV injection, a mouse restraint device was used, together with a heating lamp to dilate the tail vein. Blood was collected every two weeks by sub-mandibular vein. The SN and STR of the brains were micro-dissected for further analysis. All experiments were performed in quadruplicate.

### 2.10. Behavioral Analysis

Open-field and rotarod experiments were performed every two weeks as described previously [45]. Briefly, open-field measurements of spontaneous locomotor activity were accomplished using VersaMax (monitor RXYZCM-16, analyzer VMAUSB; AccuScan, Columbus, OH, USA) during a 10-min acquisition period following a 2-min accustimization. Behavioral coordination was assessed by RotaRod (AccuScan) performance using an acceleration protocol for three trials not exceeding 3 min. Data from VersaMax, including total distance traveled, horizontal activity, and vertical activity, as well as data from RotaRod were normalized. Specifically, each mouse was normalized to its basal score recorded at weeks 0 and 2 for each assessment. Assessments were compiled into a cumulative behavior score used for evaluating total motor function over time. Mice were individually monitored for motor activity based on cumulative score over time. Additionally, average behavior scores at early (i.e., weeks 4 and 6) and late (i.e., weeks 10 and 12) times were averaged and compared across groups. Finally, forepaw use bias was examined at 10 weeks post-injection for every mouse. Specifically, mice were placed in a cylindrical jar for 7 min and incidents where mice used each paw (separately or together) were counted using video. Percent ipsilateral forepaw usage was calculated based on the number of incidents for each mouse.

### 2.11. Western Blotting

Brain SN designated for Western blot analysis were micro-dissected and stored at −80 °C until further use. Western blot lysates from the SN were prepared in RIPA buffer with protease and phosphatase inhibitors. Specifically, RIPA buffer was added, and samples were homogenized and then sonicated for 5 min on ice (30 s on/off cycle). Protein estimation was performed using a Bradford assay. Samples of appropriate dilution were added to protein assay dye reagent concentrate (cat. no. 5000006, Bio-Rad) diluted 1:5 in nanopure water and absorbance was measured at 595 nm. A standard curve of BSA between 2–14 µg/mL was compared for quantification. Then, 25 µg per sample was added 1:1 to a 5:1 mixture of 2× Laemmli sample buffer (Cat. no. 161-0737, Bio-Rad) to beta-mercaptoethanol, or approximately 20 µL, into polyacrylamide gels (Cat. no. 4569034, Bio-Rad) for an SDS-PAGE run at 100 V constant voltage for 1 h 30 min at 4 °C in 10× Tris/Glycine/SDS buffer diluted 10× in dH2O. Following SDS-PAGE, proteins were transferred to a nitrocellulose membrane at 80 V constant voltage at 4 °C for 3 h using a transfer buffer of 10× Tris/Glycine (Cat. no. 1610734, Bio-Rad) diluted 10× in d_2_O with 20% methanol.

The membranes were blocked for 1 h in intercept blocking buffer (Cat. no. 927-70001, Li-Cor Biosciences, Lincoln, NE, USA) at room temperature. Membranes were cut into sections according to molecular weight and incubated with either anti-tyrosine hydroxylase-mouse mAb at 1:2000 (Cat. no. MAB 318, EMD Millipore, Burlington, MA, USA) or anti-αSyn mouse mAb at 1:1000 (Cat. no. 610787, BD Biosciences, San Jose, CA, USA) in blocking buffer with 0.05% T-20 for 16 h at room temperature. Membranes were washed 5× with 1× PBS + 0.05% T-20 (wash buffer), then incubated with 800CW Donkey anti-Mouse IgG secondary (Cat. no. 926-32212, Li-Cor Biosciences) 1:15000 in blocking buffer + 0.05% T-20 for 1 h at room temperature. Membranes were washed 5× in wash buffer, then imaged using a Li-Cor Odyssey infrared scanner (Model # 9120 Odyssey, Li-Cor Biosciences). Membranes were incubated with anti-beta actin-rabbit mAb (Cat. no. A2103, Sigma) 1:1000 + 680RD Donkey anti-Rabbit IgG secondary (Cat. no. 926-68073, Li-Cor Biosciences) 1:15,000 in blocking buffer + 0.05% T-20 for 1 h at room temperature. Membranes were washed 5× in wash buffer then imaged once more.

### 2.12. Dot Immunoblotting

Nitrocellulose membranes were spotted twice with 2.5 μLof 1 μg/μL αSyn_agg_, αSyn, BSA (non-specific control), or SN brain homogenate used for Western blotting (25 µg total per sample). After letting the blots dry, 5 mL of 5 wt % skim milk in 1× PBS was added to the blots inside microcentrifuge tubes, and the blots were blocked on a Labquake tube shaker/rotator (Fisher Scientific) for 60 min at room temperature (RT). The blots were rinsed three times with 1× PBS; then, 5 mL of 10 μg/mL purified singularly specific monoclonal antibody IgM 3C5 or 5H6 developed and purified in-house according to previous work in a dilution buffer of 0.1 wt % T-20 in 1× PBS, and the blots were incubated on a tube rotator for 90 min at RT [19]. The blots were rinsed three times with dilution buffer, then incubated with 800CW donkey anti-Mouse IgG secondary (Cat. no. 926-32212, Li-Cor Biosciences) 1:15,000 in dilution buffer before imaging.

### 2.13. High Performance Liquid Chromatography (HPLC) for Catecholamine Analysis

Brain STR were micro-dissected, preserved in antioxidant mixture containing 0.2 M perchloric acid, 0.1% NA2S2O5, 0.05% NaEDTA, and isoproterenol as an internal standard as described previously and immediately stored in dry ice [46]. The samples were further processed for HPLC-ECD with pump (Thermo-Scientific ISO-3100SD-BM) and autosampler (Thermo-Scientific WPS-3000 TSL) using a CoulArray 5600A-ECD detection system operated at 25 °C. Specifically, 20 µL samples were injected onto an Agilent Eclipse Plus (3.5 µm, 100A) C18 HPLC column (150 × 4.60 mm). Then, analytes were eluted with MD-TM MP mobile phase (Cat. no. 701332, Thermo Fisher Scientific) under isocratic conditions at 0.6 mL/min for 21 min. Dopamine (DA) and its major metabolites, 3,4-dihydroxyphenylacetic acid (DOPAC) and homovanillic acid (HVA), were quantified using a standard curve. All sample concentrations were corrected using isoproterenol (ISO) as an internal standard correction factor and expressed as ng neurotransmitter/mg wet tissue weight.

### 2.14. Immunohistochemistry for Fluorescent and Chromogenic Analysis

Animals were transcranially perfused with PBS and 4% PFA and stored in 4% PFA at 4 °C until all blood vessels were visibly transparent. SN and STR sections were dissected and placed in cassettes which were stored in 4% PFA for 2 d at 4 °C. Tissues were embedded in paraffin, and 5 µm slide sections were mounted onto positively charged slides by the Iowa State University clinical pathology laboratory. To process tissues for immunohistochemistry, slide sections of SN and STR were de-paraffinized by heating at 60 °C for 30 min and then using a gradient solvent method with xylene (2×), 50/50 xylene/ethanol, 100% ethanol, 90%, ethanol in water, 70% ethanol, and 50% ethanol. Following rinses in water and then 3× in PBS, antigen retrieval was achieved by keeping sections at 80 °C for 30 min in 10 mM sodium citrate buffer, pH 6.

For preparation of slides for fluorescent imaging, slides were pre-incubated for 1 h in a blocking buffer containing 10% donkey serum (DS), 2% bovine serum albumin, and 0.05% Tween-20 (T-20) in PBS. Then, slides were incubated in 2% DS and 0.05% T-20 with 1:2000 rabbit anti-tyrosine hydroxylase polyclonal antibody (Cat. No. 657012, EMD Millipore, Burlington, MA, USA) and 1:500 mouse anti-s129 αSyn (Fujifilm Wako Chemicals, Richmond, VA, USA) for 16 h at room temperature. After washing the tissues 6 × 10 min in 1× PBS, donkey anti-mouse 555 (cat. no. A-31570, Thermo Fisher Scientific) or 647 (Cat. no. A-31571, Thermo Fisher Scientific) and donkey anti-rabbit 488 (Cat. no. A-21206, Thermo Fisher Scientific) was incubated in 2% DS with 0.05% T-20 for 1 h. Sections were washed again 6 × 5 min in 1× PBS, then Hoechst (1:5000) was incubated in slides for 5 min. Slides were washed 2 × 5 min and then mounted using Fluoromount™ aqueous mounting medium (Cat. no. F4680-25ML, Sigma) and allowed to dry for at least 18–24 h.

For preparation of slides for bright-field imaging, tissues were additionally incubated in methanol + 3% hydrogen peroxide (H325-100, Thermo Fisher Scientific), 3× in 1× PBS, then pre-incubated for 1 h in a blocking buffer containing 10% normalized goat serum (NGS, Cat. no. S-1000-20, Vector Laboratories, Burlingame, CA, USA), 2% bovine serum albumin (BSA, Cat. no. A9418, Millipore Sigma, St. Louis, MO, USA), and 0.5% Triton-X in 1× PBS. Blocking buffer was removed and slides were then incubated in 2% NGS and 0.05% T-20 with 1:1600 rabbit anti-tyrosine hydroxylase polyclonal antibody for 16 h at 4 °C. After washing the tissues 6 × 5 min in 1× pbs, goat anti-rabbit (Cat. no. BA-1000, Vector Laboratories) was incubated in 2% NGS with 0.05% T-20 for 1 h. Tissues were washed 6 × 5 min in 1× PBS, then incubated with ABC solution (Cat. no. PK-6100, Vector Laboratories), which was pre-equilibrated for at least 30 min prior to incubation for 1 h at RT. Tissues were washed 3 × 5 min in PBS, then incubated with 2 mM 3,3′-diaminobenzidine + 0.005% hydrogen peroxide (DAB) in 1× PBS for 1 min until color development, then immediately exchanged for PBS and washed 4 × 5 min. Tissues were dried overnight, then dehydrated by a gradient solvent method in reverse order from that presented above, then DPX mounting medium (Cat. no. 44581, Millipore Sigma) was added to the slides and samples were covered with coverslips and allowed to dry for at least 18–24 h. Both fluorescent and brightfield images were taken using a BZ-X810 All-in-One Fluorescence Microscope (Keyence America, Itasca, IL, USA). SN and STR regions for each set of slides were imaged under the same conditions. Densitometric analysis of specified regions from fluorescence microscopic images were further analyzed by ImageJ to evaluate TH and αSyn expression.

### 2.15. Statistical Analyses

Two methods of normalization were used in motor and immunohistochemical microscopic analysis. The first normalization method involved calculating the percent of ipsilateral/contralateral control, i.e., the level of expression of proteins and neurotransmitters of mice unilaterally injected with AAV were compared to levels in the non-injected, or contralateral, site. The normalized ipsilateral/contralateral control is expressed as:

$$100\times \frac{X_{ipsilateral}}{X_{contralateral}}$$

Here, *X* is the relative protein or catecholamine expression from the same mouse for both values. The second normalization method was used only for the cumulative score behavioral data in motor and immunohistochemical microscopic analysis. Specifically, data were normalized based on basal activity for each individual assessment prior to cumulating data into a behavior score and analysis, due to differences in units across individual behavior assessments. All Western, dot blot, and immunohistochemical images were analyzed by the ImageJ densitometric analysis–gel analysis tool. Individual and cumulative score behavioral data were log-transformed prior to analysis due to high variability within sample groups. Statistical analysis was performed using GraphPad^®^ Prism software. Specifically, a one-way ANOVA using Dunnett’s multiple comparisons (two-tailed) was used for all percent ipsilateral/contralateral control comparisons. All statistical tests were set at multiple levels to denote varying degrees of significance including: * *p* ≤ 0.05, ** *p* ≤ 0.01, *** *p* ≤ 0.001, and **** *p* ≤ 0.0001. Additionally, linear mixed models (LMMs) were used for analyzing the repeated measures behavioral data using R coding software. Further, the Benjamini–Hoch method was used to correct for multiple comparisons in the behavioral data. All comparisons denoting statistical significance was defined as *p* < 0.05.

Sample sizes for each experiment were as follows: SEM—n = 4 images counting 100 particles per image; zeta potential and release—n = 3; biodistribution—n = 4 per group, per time point; animal behavior—n = 8 for scFv and NP, n = 7 for AAV, n = 6 for control; catecholamine and dot and Western blot analysis—n = 4 for AAV, scFv and NP, n = 3 for control; immunohistochemistry (brightfield and fluorescence) analysis—n= 3 for control and AAV, n = 4 for scFv and NP. No outliers were excluded from the datasets. All release, dot, and Western blot individual behavior assessment data are presented in motor and immunohistochemical microscopic analysis; catecholamine and immunohistochemistry data are presented as mean ± SEM. Cumulative behavior score data in motor and immunohistochemical microscopic analysis and individual behavioral assessment data in Supplementary Figure S1 are expressed as median ± 95% confidence interval.

## 3. Results

### 3.1. scFv, Polymer, NP Characterization and Drug Release Kinetics

**Figure 1:**
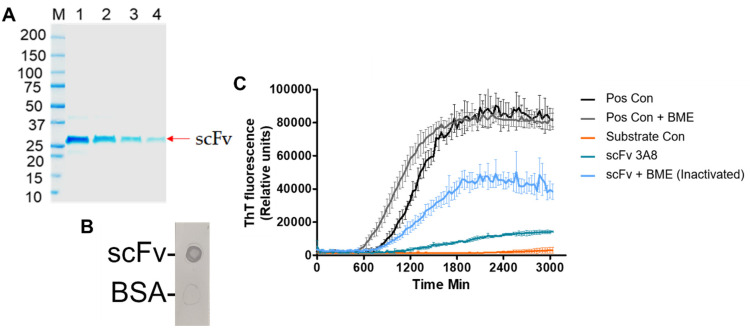
Validation of scFv for therapeutic studies. (**A**) SDS-PAGE of purified scFv indicates a molecular weight of 27 kDa. (**B**) Anti-myc tag dot immunoblot of scFv confirming activity of scFv. (**C**) RT-QuIC assay of scFv. Pos control: monomeric, recombinant αSyn incubated with PD brain homogenates to seed fibril formation. Substrate control: reaction mixture incubated without brain homogenates to ensure aggregation was caused by brain homogenates only. scFv 3A8: monomeric, recombinant αSyn incubated with PD brain homogenates + scFv to inhibit fibril formation. scFv + BME (inactivated): scFv-inactivated with 1mM BME final concentration prior to adding to wells to ensure reduction in aggregation was associated with scFv 3A8-specific activity.

The purified scFv had a molecular weight of approximately 27 kDa and displayed activity against anti-myc tag (Figure 1A,B). An RT-QuIC assay of scFv incubated with recombinant αSyn indicated that scFv suppressed aggregation in vitro (Figure 1C). 

**Figure 2:**
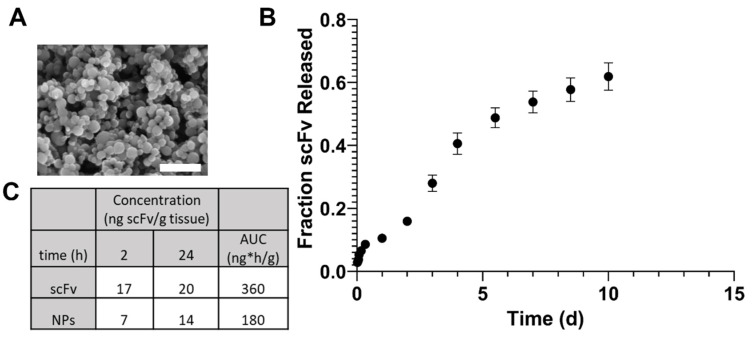
NP morphology and release profile. (**A**) SEM photomicrograph of scFv-encapsulated NPs indicates discrete morphology with a geometric mean ± SD of 345 ± 1.28 nm. Zeta potential mean ± S.E. of −32.1 mV ± 0.7 mV. Scale bar: 2 µm. (**B**) scFv release profile from NP encapsulation. Following a burst release in the first six hours, a sustained release profile was observed over 10 days, with an encapsulation efficiency of 27%. (**C**) Ex vivo tissue distribution of soluble scFv or NPs in the brain over time. Mass scFv in ng normalized to tissue mass (mg).

10:90 CPTEG:SA copolymer in molecular weight range of 15,000–20,000 g/mol was used for synthesizing NPs loaded with scFv, as demonstrated previously [47]. Additionally, NP suspension quality, morphology, size, and polydispersity were within standard ranges of previously reported values for the chemistries and procedures used (Figure 2A) [38,41,43]. The NPs displayed a 10% burst release of scFv followed by sustained release of scFv over seven days, with an encapsulation efficiency of 27% (Figure 2B).

### 3.2. Soluble scFv NPs Detected in the Brain after IV Administration

After quantifying fluorescence intensity, it was found that the mass of scFv in the brain remained constant for one day after IV administration (Figure 2C). However, no significant differences in the concentration of scFv were observed between the soluble scFv and scFv-encapsulated NP treatments. Specifically, scFV concentration in the brain upon administration of a soluble dose was 17 ng/mg and 20 ng/mg of tissue at 2 h and 1 d after administration, respectively, and that of scFv-encapsulated NPs was 7 ng/mg and 14 ng/mg tissue, respectively.

### 3.3. Motor Activity Recovered with scFv Treatment

**Figure 3:**
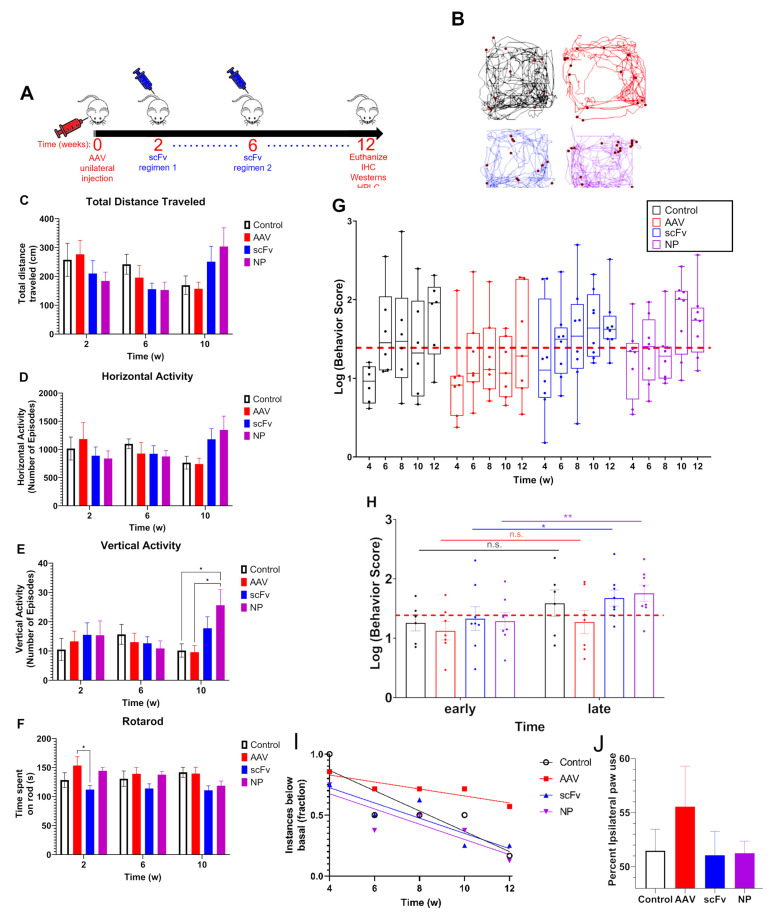
Motor deficits observed from AAV injection are recovered with scFv treatment. (**A**) Mice were injected with AAV at time t = 0, followed by delayed scFv treatment beginning at t = 2 weeks. scFv treatment by lateral tail vein injection was performed 3× weekly until week 6 (regimen 1), at which point injections were performed 1× weekly at a higher dose (regimen 2). (**B**) Versa plot showing representative horizontal and vertical (red dots) activity of control (bottom left), AAV (top right), scFv (top left), and NP (bottom right) groups at 12 weeks post AAV injection. Spontaneous locomotor activity, including total distance traveled (**C**), horizontal activity (**D**), vertical activity (**E**), and coordination by rotarod test (**F**) were individually assessed for each mouse. Any comparisons not denoted are not significant with *p* ≥ 0.05. Statistical tests include ordinary two-way ANOVA with Tukey’s multiple comparisons (two-tailed). N = 3 control; n = 4 AAV, scFv, NP. Data presented as mean ± SEM. (**G**) Spontaneous locomotor activity, including total distance traveled, and horizontal and vertical activity, as well as coordination by rotarod test were individually assessed for each mouse. Results were compared to basal recordings (i.e., average of 0 and 2 weeks) and compiled into a cumulative behavior score. (**H**) Cumulative behavior scores of mice at early (i.e., weeks 4 and 6) as well as late (i.e., weeks 10 and 12) time points. Statistical significance generated by a linear mixed model comparing early vs. late behavior scores within each group is shown by * *p* ≤ 0.05, ** *p* ≤ 0.01. (**I**) Fraction of mice within each group at each time point displaying cumulative motor performance below basal (i.e., average of weeks 0 + 2). Linear trends show improvement in behavior scores among control, scFv, and NP groups, with week 12 indicating a fraction of 0.167, 0.25, and 0.125 of mice displaying motor activity below basal, but less so in AAV groups, with a fraction 0.571 of mice displaying motor activity below basal. (**J**) Forepaw use preference at 10 weeks.

After unilateral AAV injection, mice were monitored for a decrease in motor function over 12 weeks (Figure 3A). Representative 2D versa plots of mice from each group at 12 weeks post-injection indicate slight differences in total motor activity (Figure 3B). Spontaneous locomotor activity such as total distance traveled (Figure 3C), horizontal activity (Figure 3D), vertical activity (Figure 3E), and coordination by rotarod test (Figure 3F) were individually assessed for each mouse and statistically significant differences with *p* < 0.05 are indicated. After normalizing and compiling all individual assessments, a trend indicating an overall decrease in motor function over time in mice injected with AAV without treatment compared to PBS control was observed (Figure 3G). However, scFv-based treatments recovered behavioral activity in most mice receiving either soluble scFv or NP-encapsulated scFv formulations (Figure 3H). Linear mixed models were used to statistically assess repeated measures data generated by bi-weekly motor activity assessment. Differences between the average of early (i.e., weeks 4 and 6) and late (i.e., weeks 10 and 12) time points within groups were compared (Figure 3H). It was found that the behavioral activity at later times was significantly higher than that at early times in the scFv and NP-encapsulated groups. In contrast, no significant differences were observed in the animals that received AAV treatment only (Figure 3H).

This was also correlated with overall poorer activity at both early and late time points among AAV-only mice, where activity did not recover to basal levels in most of these animals. In contrast, mice that received either scFv treatment (i.e., soluble or NP-encapsulated) displayed a trend of recovered motor performance to basal levels, particularly at later times (Figure 3I). Specifically, at week 12, only 16.7%, 25%, and 12.5% of mice in the control, scFv, and NP groups, respectively, displayed motor activity below basal levels. In contrast, 57.1% of AAV-only mice displayed motor activity below basal levels (Figure 3I). Contrast differences across groups within weeks were also compared. There were no significant differences observed, except in the case of AAV and NP-encapsulated scFv treatments, which approached significance at 10 weeks (*p* = 0.095). The forepaw use studies did not show statistically significant differences between the groups (Figure 3J).

### 3.4. Protein Expression and Catecholamine Expression Analysis

Western blots were performed to assess immunoreactivity of αSyn and TH in the SN 12 weeks after AAV injection (Supplementary Figures S1 and S2). Due to unilateral injection, the contralateral, or non-injected site of the mice was compared to the ipsilateral site, for TH expression and α-Syn expression (Supplementary Figure S3A,B). Soluble and NP-encapsulated scFv treatments displayed a trend of reducing αSyn in the injected site, but no significant differences were observed (Supplementary Figure S3B). This lack of significant neurodegeneration in the AAV model after 12 weeks has been observed in other studies in the literature [12,13]. Similar trends were observed in the expression of αSyn_agg_ in AAV-treated mice in a dot immunoblot (Supplementary Figure S3C), regardless of treatment. STR tissue sections were processed for HPLC analysis of neurotransmitters. Neither the levels of dopamine nor 3,4-dihydroxyphenylacetic acid (DOPAC) in the STR, with percent ipsilateral/contralateral controls for the different groups, showed statistically significant differences (Supplementary Figure S3D,E).

### 3.5. Immunohistochemical Analysis

**Figure 4:**
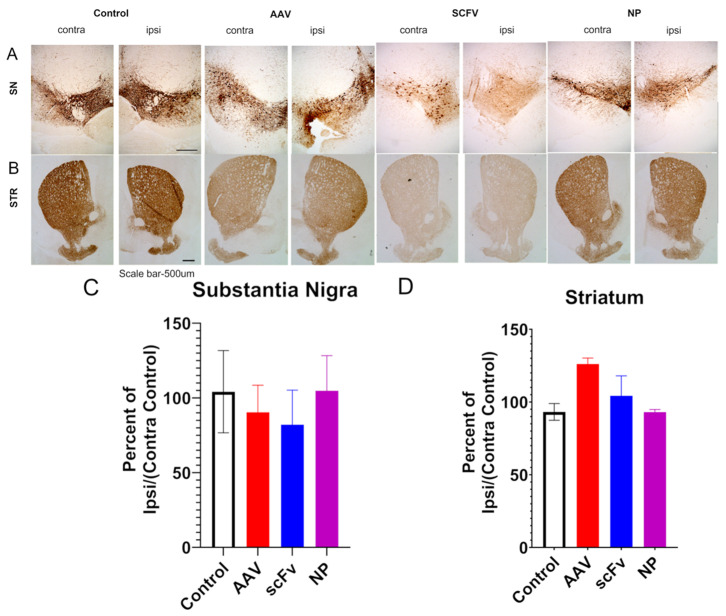
Immunohistochemical brightfield microscopic analysis. (**A**) Representative SN images of control, AAV, scFv, and NP mice. Scale bar 500 µm. (**B**) Representative STR images of control, AAV, scFv, and NP mice. Scale bar 500 µm. (**C**) Densitometric analysis of TH expression in SN showing percent ipsilateral/contralateral control change over contralateral control. (**D**) Densitometric analysis of TH expression in STR showing percent ipsilateral/contralateral control change over contralateral control.

**Figure 5:**
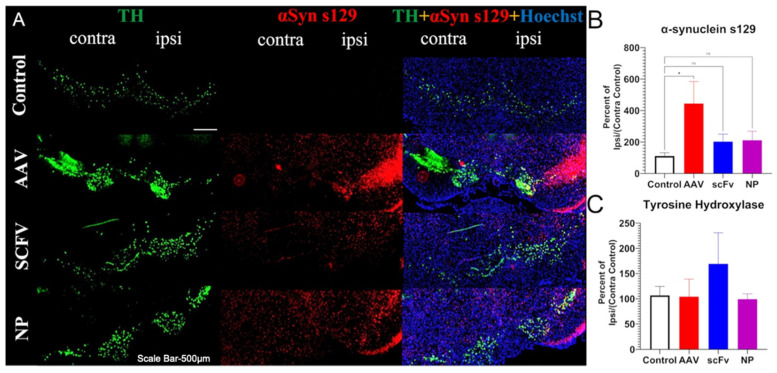
αSynuclein s129 and TH immunohistochemical fluorescent microscopic analysis in SN indicates increase in percent ipsi/(contra control) αSyn expression after unilateral AAV injection, which is ameliorated by scFv treatment. (**A**) TH, αSyn s129, and merged (+Hoechst) representative fluorescent images in the SN of control, AAV, scFv, and NP. Scale bar 500 µm. (**B**) Densitometric analysis of αSyn s129 in the SN showing percent ipsi/(contra control) of αSyn. (**C**) Densitometric analysis of TH in the SN showing percent ipsi/(contra control). In statistical comparisons, * *p* ≤ 0.05.

It was found that AAV injection did not show a statistically significant decrease in TH immunoreactivity expressed as a percentage of ipsilateral/contralateral controls over the 12-week timeframe of this study in SN or STR (Figure 4). These results are consistent with other studies in the literature with this PD model that indicate that neurodegeneration takes a few months after the AAV injections [12,13]. However, immunohistochemical fluorescence microscopic analysis indicated that the AAV injection significantly increased αSyn s129 expression in terms of the percent of ipsilateral/contralateral control in the SN (Figure 5B). In contrast, in animals treated with soluble scFv and NP-encapsulated scFv, the percent ipsilateral/contralateral control of αSyn s129 expression levels decreased compared to the AAV positive control and was similar to the negative control group (Figure 5B). These trends were not as apparent in the TH expression levels (Figure 5C), consistent with the immunohistochemical analysis in Figure 4C. There were no significant differences seen in STR.

## 4. Discussion

Due to the prevalence of αSyn mutations and subsequent aggregation in PD pathology, disease-slowing therapeutics for PD must be able to address this underlying mechanism. To this end, recombinant protein and antibody-based therapeutics represent a promising approach to ameliorating αSyn over-expression through specific binding to αSyns129 and αSyn_agg_. This is particularly important due to the importance of physiological αSyn for neuronal homeostasis. The scFvs developed in our previous work [19] with high specificity for αSyn_agg_ may therefore be able to provide therapeutic efficacy in an αSyn over-expression model of PD.

After expression and purification of scFv in *E. coli*, scFv was assessed for activity and efficacy. An SDS-PAGE of the scFv indicated a molecular weight of 27 kDa (Figure 1A), which agrees with the average reported molecular weight of scFvs in literature [48]. Additionally, the activity of the scFv was confirmed by an anti-myc tag dot blot (Figure 1B). Finally, an RT-QuIC assay was performed to evaluate the efficacy of the scFv (Figure 1C). By reducing the extent of aggregation promoted by recombinant αSyn, in vitro efficacy of the scFv was confirmed. These results are also in agreement with previous work, indicating the robustness of the scFv in providing therapeutic efficacy in vitro [19].

In order to potentially enhance therapeutic efficacy in vivo, scFv was encapsulated within polyanhydride NPs. These NPs produced standard morphology and size for SA-rich chemistries, indicating the feasibility of encapsulation (Figure 2A). The release profile of scFv showed a small burst, followed by a sustained release for one week (Figure 2B). The burst release could allow for rapid treatment of AAV-induced αSyn over-expression, while the sustained release can help maintain higher bioavailability for a longer time between doses.

In a preliminary biodistribution study, brain bioavailability of scFv after soluble and NP administration IV showed that there were no significant differences in scFv concentration within one day (Figure 2C). However, our previous studies have shown that SA-rich NP formulations lead to greater percentages of brain bioavailability at later (i.e., 3–7 days) time points [40]. Additionally, the 2 h and 1 d brain bioavailability profiles for fluorescent dye in that study showed similar trends to that with scFv in the current studies, suggesting that the NPs can provide sustained bioavailability of therapeutics over longer periods of time than what can be achieved with a soluble dose [40].

Behavioral deficits are fundamental characteristics of later-stage PD. By performing behavioral assessments, we sought to determine whether the observed brain bioavailability of scFv in our previous biodistribution studies would translate to recovery of motor activity when mice are injected with AAV. Although some dopaminergic neuronal loss is known to occur after AAV injection within a time period of 6–16 weeks for this model (and similar models), a 25% reduction in dopaminergic neurons was seen only 6 months after transduction [12,13]. Hence, this shorter time period of the study may not have been sufficient to see more pronounced behavioral deficits [10,11,49,50]. However, a previous study evaluating this model in mice evaluated nigrostriatal degeneration 10 weeks post-injection and observed behavioral deficits based on forepaw preference using the cylinder test as early as six weeks post-injection [15].

Due to the various individual assessments and variability that is commonly observed between mice in each group, behavioral data can become convoluted. Therefore, we used a cumulative behavior score that compiled normalized data from four separate behavioral assessments (total distance traveled, horizontal activity, vertical activity, and time spent on rotarod), all of which are expected to be affected by AAV injection, into a single value. The rationale for this inclusion is to allow for a comprehensive evaluation of the total motor impairment at any given time throughout the course of the study.

In our studies, a slightly longer endpoint of 12 weeks post-injection was chosen to allow time for a more pronounced decrease in motor activity. It was found from VersaMax and rotarod behavioral assessments that there was a trend of consistent decrease in motor activity over time among AAV-injected mice, suggesting that this model can produce rapid behavioral deficits. Basal activity was assessed based on the average of 0–2 weeks data before initiation of treatment. Both time points were used for basal data as no significant differences were observed in behavioral activity between 0–2 weeks after AAV injection. Additionally, using two time points for measuring activity allowed for more robust statistical analysis of trends in behavior using the LMM discussed above. It was found that after an initial depression in activity across all groups at week 4, the scFv-treated groups (as well as control) recovered back to basal activity. In contrast, animals that received only AAV (i.e., no treatments) did not recover (Figure 3). Forepaw use bias further corroborated this observation of a trend indicating that AAV positive control mice did not recover motor activity as well as mice treated with scFv solubly or via NP-encapsulation. Because dopaminergic neurons in the nigrostriatal pathway are responsible for controlling motor coordination [51], it appears that both soluble scFv and NP-encapsulated scFv groups protected against αSyn over-expression in the SN of these animals.

To support this finding, immunochemical analyses were performed on the SN and STR. Some previous work has shown that this AAV1/2-A53T model can successfully produce rapid nigrostriatal degeneration in C57BL/6 mice [15]. Other studies have indicated that neurodegeneration takes longer in this AAV model but that the overexpression of α-synuclein alone, even without the neurodegeneration is sufficient to trigger microglial activation and neuroinflammation, as seen in PD [12,13]. No significant decreases in the TH of AAV-injected mice were observed, in agreement with these previous findings [12,13]. In addition, no significant differences in levels of dopamine or of 3,4-dihydroxyphenylacetic acid, a metabolite of dopamine were seen in the STR, suggesting that this model did not produce pronounced nigrostriatal degeneration in 12 weeks.

In addition to TH loss, because this is an αSyn over-expression model, the levels of αSyn were expected to be higher in the ipsilateral SN of injected mice. The exact mechanisms of αSyn pathology in PD are still not fully known, because protein aggregation plays a role in many neurodegenerative diseases [18]. What is known is that the phosphorylation of the 129th residue of αSyn increases the propensity for aggregation and subsequently other pathological characteristics, which can lead to malfunctions in synaptic activity that ultimately impedes neuronal function [52]. To thoroughly evaluate the extent of pathology, it is therefore important to evaluate not only the expression of physiological αSyn, but also phosphorylated (s129) αSyn and αSyn_agg_, because each form provides unique insights into the mechanisms at play and the extent of disease.

In our studies, a pronounced increase in αSyns129 levels was observed after AAV injection (Figure 5B). Because αSyns129 is thought to induce oligomeric αSyn followed by αSyn_agg_ in early-stage disease, this may indicate that the mice were at an early stage of the degenerative process [53]. Both soluble scFv and NP-encapsulated scFv formulations protected animals against over-expression of physiological αSyn (Figure 5B). In future studies, further optimization, including larger doses of the scFv and changing the route of administration of the scFv could also improve therapeutic efficacy and distinguish between the efficacy of soluble versus NP-loaded scFV. For example, larger and more frequent doses can be administered by the less-invasive intraperitoneal delivery method, albeit with a slower rate of absorption [50] and could potentially be used in combination with the more rapid effects induced by IV treatment [54].

The scFv described in this preliminary study, which is specific exclusively for pathological aggregates of αSyn, shows early promise in blocking PD progression in a surrogate mouse PD model, although further studies will be needed to definitively determine its use as a therapeutic agent in PD patients. Especially encouraging is the recent accelerated approval by the United States Federal and Drug Administration (FDA) of Aduhelm (Aducanumab) developed by Biogen for management of Alzheimer’s disease (AD) [55], a progressive and debilitating neurodegenerative disease affecting 6.2 million Americans currently. Much like the scFv described in this study, Aducanumab, a high affinity, human IgG1 monoclonal antibody is directed against a conformational epitope uniquely on amyloid beta (Aβ) aggregates/plaques (but not the monomer) that are pathognomic of AD [56]. Aducanumab significantly reduced levels of Aβ in the brains of AD patients in two phase III clinical trials [55], which strongly suggests that this drug will likely block disease progression and prevent further deterioration, thereby facilitating the maintenance of the quality of life of AD patients.

## 5. Conclusions

There is an urgent unmet need for novel and efficacious preventive and therapeutic management modalities for degenerative neurological disorders, including PD. It is imperative that such a therapeutic management blocks PD progression without eliciting toxic systemic deleterious effects. Among disease-slowing treatment options, anti-αSyn-targeting antibody therapeutics are becoming a well-studied area of research due to the prevalence of αSyn pathology in PD. As a prelude to in vivo determination of therapeutic potential, we first validated the robustness of the AAV1/2-A53T model in mice, which showed similar pathology to that shown in previous studies. Then, by assessing motor activity and protein, protection against SN AAV injection was observed in animals receiving both soluble scFV and NP-encapsulated scFv treatments. These scFv-based formulations and potential cocktail treatment regimens combining soluble and nanoparticle-encapsulated scFv should be further optimized and evaluated in additional proxy animal models to assess their use as a therapeutic modality to slow down or halt PD progression.

## Data Availability

Data available on request.

## Supplementary Materials

The following supporting information can be downloaded at: https://www.mdpi.com/article/10.3390/biom13081203/s1, Figure S1. Western blot images taken of the ipsilateral (ipsi) and contralateral (contra) SN in mice 12 weeks post AAV injection and compared to PBS-injected control. Images were used for densitometric analysis in ImageJ; Figure S2. Western blot original membranes. (A–D) lower green band: TH. Red band: B-actin. (E–H) lower green band: aSyn. Red band: B-actin.; Figure S3. (A) Densitometric analysis of relative αSyn expression levels in the SN expressed as % ipsilateral/contralateral control (B) densitometric analysis of relative TH expression levels in the SN expressed as % ipsilateral/contralateral control (C) DA concentration in ng/mg in the STR, evaluated by HPLC, expressed as % ipsilateral/contralateral control (D) DOPAC concentration in ng/mg in the STR, evaluated by HPLC, expressed as % ipsilateral/contralateral control. (E) Representative dot blot images of samples taken from SN and evaluated for αSyn_agg_ expression by dot immunoblotting.

## References

[1] Dauer W., Przedborski S. (2003). Parkinson’s disease: Mechanisms and models. Neuron.

[2] De Luca C.M.G., Elia A.E., Portaleone S.M., Cazzaniga F.A., Rossi M., Bistaffa E., De Cecco E., Narkiewicz J., Salzano G., Carletta O. (2019). Efficient RT-QuIC seeding activity for alpha-synuclein in olfactory mucosa samples of patients with Parkinson’s disease and multiple system atrophy. Transl. Neurodegener..

[3] Branco D.M., Arduino D.M., Esteves A.R., Silva D.F., Cardoso S.M., Oliveira C.R. (2010). Cross-talk between mitochondria and proteasome in Parkinson’s disease pathogenesis. Front. Aging Neurosci..

[4] Sato H., Kato T., Arawaka S. (2013). The role of Ser129 phosphorylation of alpha-synuclein in neurodegeneration of Parkinson’s disease: A review of in vivo models. Rev. Neurosci..

[5] Polymeropoulos M.H., Lavedan C., Leroy E., Ide S.E., Dehejia A., Dutra A., Pike B., Root H., Rubenstein J., Boyer R. (1997). Mutation in the alpha-synuclein gene identified in families with Parkinson’s disease. Science.

[6] Fuchs J., Nilsson C., Kachergus J., Munz M., Larsson E.M., Schule B., Langston J.W., Middleton F.A., Ross O.A., Hulihan M. (2007). Phenotypic variation in a large Swedish pedigree due to SNCA duplication and triplication. Neurology.

[7] Chesselet M.F., Richter F. (2011). Modelling of Parkinson’s disease in mice. Lancet Neurol..

[8] Van der Perren A., Toelen J., Carlon M., Van den Haute C., Coun F., Heeman B., Reumers V., Vandenberghe L.H., Wilson J.M., Debyser Z. (2011). Efficient and stable transduction of dopaminergic neurons in rat substantia nigra by rAAV 2/1, 2/2, 2/5, 2/6.2, 2/7, 2/8 and 2/9. Gene Ther..

[9] Taymans J.M., Vandenberghe L.H., Haute C.V., Thiry I., Deroose C.M., Mortelmans L., Wilson J.M., Debyser Z., Baekelandt V. (2007). Comparative analysis of adeno-associated viral vector serotypes 1, 2, 5, 7, and 8 in mouse brain. Hum. Gene Ther..

[10] Koprich J.B., Johnston T.H., Reyes M.G., Sun X., Brotchie J.M. (2010). Expression of human A53T alpha-synuclein in the rat substantia nigra using a novel AAV1/2 vector produces a rapidly evolving pathology with protein aggregation, dystrophic neurite architecture and nigrostriatal degeneration with potential to model the pathology of Parkinson’s disease. Mol. Neurodegener..

[11] Koprich J.B., Johnston T.H., Huot P., Reyes M.G., Espinosa M., Brotchie J.M. (2011). Progressive neurodegeneration or endogenous compensation in an animal model of Parkinson’s disease produced by decreasing doses of alpha-synuclein. PLoS ONE.

[12] Theodore S., Cao S., McLean P.J., Standaert D.G. (2008). Targeted overexpression of human alpha-synuclein triggers microglial activation and an adaptive immune response in a mouse model of Parkinson disease. J. Neuropathol. Exp. Neurol..

[13] St Martin J.L., Klucken J., Outeiro T.F., Nguyen P., Keller-McGandy C., Cantuti-Castelvetri I., Grammatopoulos T.N., Standaert D.G., Hyman B.T., McLean P.J. (2007). Dopaminergic neuron loss and up-regulation of chaperone protein mRNA induced by targeted over-expression of alpha-synuclein in mouse substantia nigra. J. Neurochem..

[14] Musacchio T., Rebenstorff M., Fluri F., Brotchie J.M., Volkmann J., Koprich J.B., Ip C.W. (2017). Subthalamic nucleus deep brain stimulation is neuroprotective in the A53T alpha-synuclein Parkinson’s disease rat model. Ann. Neurol..

[15] Ip C.W., Klaus L.C., Karikari A.A., Visanji N.P., Brotchie J.M., Lang A.E., Volkmann J., Koprich J.B. (2017). AAV1/2-induced overexpression of A53T-alpha-synuclein in the substantia nigra results in degeneration of the nigrostriatal system with Lewy-like pathology and motor impairment: A new mouse model for Parkinson’s disease. Acta Neuropathol. Commun..

[16] Kim D., Kim Y.S., Shin D.W., Park C.S., Kang J.H. (2016). Harnessing Cerebrospinal Fluid Biomarkers in Clinical Trials for Treating Alzheimer’s and Parkinson’s Diseases: Potential and Challenges. J. Clin. Neurol..

[17] Schlichtmann B.W., Hepker M., Palanisamy B.N., John M., Anantharam V., Kanthasamy A.G., Narasimhan B., Mallapragada S.K. (2021). Nanotechnology-Mediated Therapeutic Strategies against Synucleinopathies in Neurodegenerative Disease. Curr. Opin. Chem. Eng..

[18] Assêncio F.R. (2019). Alpha-synuclein as therapeutic target in Parkinson’s disease. Neuroforum.

[19] Schlichtmann B.W., Kondru N., Hepker M.M., Kanthasamy A.G., Anantharam V., John M., Ban B., Mallapragada S.K., Narasimhan B. (2020). Enzyme Immunoassay-Based Platform for Accurate Detection of Serum Pathological alpha-Synuclein in Parkinson’s Disease Patients. ACS Chem. Neurosci..

[20] Narasimhan B., Mallapragada S., John M., Kanthasamy A., Anantharam V. (2023). Alpha-Synuclein-Based Nano-Theranostics for Parkinson’s Disease. 2019. U.S. Patent.

[21] Ross K.A., Brenza T.M., Binnebose A.M., Phanse Y., Kanthasamy A.G., Gendelman H.E., Salem A.K., Bartholomay L.C., Bellaire B.H., Narasimhan B. (2015). Nano-enabled delivery of diverse payloads across complex biological barriers. J. Control. Release.

[22] Mallapragada S.K., Brenza T.M., McMillan J.M., Narasimhan B., Sakaguchi D.S., Sharma A.D., Zbarska S., Gendelman H.E. (2015). Enabling nanomaterial, nanofabrication and cellular technologies for nanoneuromedicines. Nanomedicine.

[23] Gendelman H.E., Anantharam V., Bronich T., Ghaisas S., Jin H., Kanthasamy A.G., Liu X., McMillan J., Mosley R.L., Narasimhan B. (2015). Nanoneuromedicines for degenerative, inflammatory, and infectious nervous system diseases. Nanomedicine.

[24] Kumar N., Langer R.S., Domb A.J. (2002). Polyanhydrides: An overview. Adv. Drug Deliv. Rev..

[25] Katti D.S., Lakshmi S., Langer R., Laurencin C.T. (2002). Toxicity, biodegradation and elimination of polyanhydrides. Adv. Drug Deliv. Rev..

[26] Domb A., Heller J., Kumar N. (2002). Polyanhydrides and Poly(ortho esters). Adv. Drug Deliv. Rev..

[27] Jain J.P., Modi S., Kumar N. (2008). Hydroxy fatty acid based polyanhydride as drug delivery system: Synthesis, characterization, in vitro degradation, drug release, and biocompatibility. J. Biomed. Mater. Res. A.

[28] Westphal M., Ram Z., Riddle V., Hilt D., Bortey E., Executive Committee of the Gliadel Study Group (2006). Gliadel wafer in initial surgery for malignant glioma: Long-term follow-up of a multicenter controlled trial. Acta Neurochir..

[29] Von Burkersroda F., Schedl L., Gopferich A. (2002). Why degradable polymers undergo surface erosion or bulk erosion. Biomaterials.

[30] Gopferich A., Tessmar J. (2002). Polyanhydride degradation and erosion. Adv. Drug Deliv. Rev..

[31] Tabata Y., Langer R. (1993). Polyanhydride microspheres that display near-constant release of water-soluble model drug compounds. Pharm. Res..

[32] Shieh L., Tamada J., Chen I., Pang J., Domb A., Langer R. (1994). Erosion of a new family of biodegradable polyanhydrides. J. Biomed. Mater. Res..

[33] Larobina D., Kipper M.J., Mensitieri G., Narasimhan B. (2002). Mechanistic understanding of degradation in bioerodible polyanhydrides: Consequences for drug delivery. AIChE J..

[34] Manoharan C., Singh J. (2009). Evaluation of polyanhydride microspheres for basal insulin delivery: Effect of copolymer composition and zinc salt on encapsulation, in vitro release, stability, in vivo absorption and bioactivity in diabetic rats. J. Pharm. Sci..

[35] Phanse Y., Lueth P., Ramer-Tait A.E., Carrillo-Conde B.R., Wannemuehler M.J., Narasihan B., Bellaire B.H. (2016). Cellular Internalization Mechanisms of Polyanhydride Particles: Implications for Rational Design of Drug Delivery Vehicles. J. Biomed. Nanotechnol..

[36] Binnebose A.M., Haughney S.L., Martin R., Imerman P.M., Narasimhan B., Bellaire B.H. (2015). Polyanhydride Nanoparticle Delivery Platform Dramatically Enhances Killing of Filarial Worms. PLoS Negl. Trop. Dis..

[37] Huntimer L., Wilson Welder J.H., Ross K., Carrillo-Conde B., Pruisner L., Wang C., Narasimhan B., Wannemuehler M.J., Ramer-Tait A.E. (2013). Single immunization with a suboptimal antigen dose encapsulated into polyanhydride microparticles promotes high titer and avid antibody responses. J. Biomed. Mater. Res. B Appl. Biomater..

[38] Brenza T.M., Ghaisas S., Ramirez J.E.V., Harischandra D., Anantharam V., Kalyanaraman B., Kanthasamy A.G., Narasimhan B. (2017). Neuronal protection against oxidative insult by polyanhydride nanoparticle-based mitochondria-targeted antioxidant therapy. Nanomedicine.

[39] Kelly S.M., Mitra A., Mathur S., Narasimhan B. (2020). Synthesis and Characterization of Rapidly Degrading Polyanhydrides as Vaccine Adjuvants. ACS Biomater. Sci. Eng..

[40] Schlichtmann B.W., Anantharam V., Kanthasamy A.G., Mallapragada S.K., Narasimhan B. (2023). Brain Bioavailability of Polyanhydride Nanoparticle Formulations.

[41] Brenza T.M., Schlichtmann B.W., Bhargavan B., Vela Ramirez J.E., Nelson R.D., Panthani M.G., McMillan J.M., Kalyanaraman B., Gendelman H.E., Anantharam V. (2018). Biodegradable polyanhydride-based nanomedicines for blood to brain drug delivery. J. Biomed. Mater. Res. A.

[42] Manne S., Kondru N., Hepker M., Jin H., Anantharam V., Lewis M., Huang X., Kanthasamy A., Kanthasamy A.G. (2019). Ultrasensitive Detection of Aggregated alpha-Synuclein in Glial Cells, Human Cerebrospinal Fluid, and Brain Tissue Using the RT-QuIC Assay: New High-Throughput Neuroimmune Biomarker Assay for Parkinsonian Disorders. J. Neuroimmune Pharmacol..

[43] Schlichtmann B.W., Kalyanaraman B., Schlichtmann R.L., Panthani M.G., Anantharam V., Kanthasamy A.G., Mallapragada S.K., Narasimhan B. (2022). Functionalized polyanhydride nanoparticles for improved treatment of mitochondrial dysfunction. J. Biomed. Mater. Res. B Appl. Biomater..

[44] Paxinos G., Franklin K. (2019). Paxinos and Franklin’s the Mouse Brain in Stereotaxic Coordinates.

[45] Ghosh A., Kanthasamy A., Joseph J., Anantharam V., Srivastava P., Dranka B.P., Kalyanaraman B., Kanthasamy A.G. (2012). Anti-inflammatory and neuroprotective effects of an orally active apocynin derivative in pre-clinical models of Parkinson’s disease. J. Neuroinflamm..

[46] Ghosh A., Langley M.R., Harischandra D.S., Neal M.L., Jin H., Anantharam V., Joseph J., Brenza T., Narasimhan B., Kanthasamy A. (2016). Mitoapocynin Treatment Protects Against Neuroinflammation and Dopaminergic Neurodegeneration in a Preclinical Animal Model of Parkinson’s Disease. J. Neuroimmune Pharmacol..

[47] Ulery B.D., Phanse Y., Sinha A., Wannemuehler M.J., Narasimhan B., Bellaire B.H. (2009). Polymer chemistry influences monocytic uptake of polyanhydride nanospheres. Pharm. Res..

[48] Mi P., Cabral H., Kataoka K. (2020). Ligand-Installed Nanocarriers toward Precision Therapy. Adv. Mater..

[49] Kirik D., Annett L.E., Burger C., Muzyczka N., Mandel R.J., Bjorklund A. (2003). Nigrostriatal alpha-synucleinopathy induced by viral vector-mediated overexpression of human alpha-synuclein: A new primate model of Parkinson’s disease. Proc. Natl. Acad. Sci. USA.

[50] Eslamboli A., Romero-Ramos M., Burger C., Bjorklund T., Muzyczka N., Mandel R.J., Baker H., Ridley R.M., Kirik D. (2007). Long-term consequences of human alpha-synuclein overexpression in the primate ventral midbrain. Brain.

[51] Poovaiah N., Davoudi Z., Peng H., Schlichtmann B., Mallapragada S., Narasimhan B., Wang Q. (2018). Treatment of neurodegenerative disorders through the blood-brain barrier using nanocarriers. Nanoscale.

[52] Kim W.S., Kagedal K., Halliday G.M. (2014). Alpha-synuclein biology in Lewy body diseases. Alzheimer’s Res. Ther..

[53] Majbour N.K., Vaikath N.N., van Dijk K.D., Ardah M.T., Varghese S., Vesterager L.B., Montezinho L.P., Poole S., Safieh-Garabedian B., Tokuda T. (2016). Oligomeric and phosphorylated alpha-synuclein as potential CSF biomarkers for Parkinson’s disease. Mol. Neurodegener..

[54] Turner P.V., Brabb T., Pekow C., Vasbinder M.A. (2011). Administration of substances to laboratory animals: Routes of administration and factors to consider. J. Am. Assoc. Lab. Anim. Sci..

[55] Dhillon S. (2021). Aducanumab: First Approval. Drugs.

[56] Sevigny J., Chiao P., Bussiere T., Weinreb P.H., Williams L., Maier M., Dunstan R., Salloway S., Chen T., Ling Y. (2016). The antibody aducanumab reduces Abeta plaques in Alzheimer’s disease. Nature.

