# Supplementary material for: Aggregation-Inhibiting scFv-Based Therapies Protect Mice against AAV1/2-Induced A53T-α-Synuclein Overexpression
Source: Biomolecules. 2023 Jul 31;13(8):1203. doi: 10.3390/biom13081203 (PMC10452369; doi:10.3390/biom13081203)
Supplement: Supplementary file 1 [file biomolecules-13-01203-s001.zip › biomolecules-2457539-supplementary.pdf]

Supporting Information

Aggregation-inhibiting scFv-based therapies protect mice against AAV1/2-induced A53T-α-synuclein overexpression

Benjamin W. Schlichtmann[1,5], Bharathi N Palanisamy[2], Emir Malovic[2], Susheel K. Nethi[1,5], Piyush Padhi[2], Monica Hepker[2], Joseph Wurtz[2], Manohar John[4,5], Bhupal Ban[6], Vellareddy Anantharam[2,3,5], Anumantha G. Kanthasamy[2,3,5], Surya Mallapragada[1,5,], and Balaji Narasimhan[1,5,*]*

Supplementary Data

RNA Isolation and cDNA Synthesis

Total RNA was isolated from cryopreserved hybridomas using a one-step RNeasy Kit (Qiagen; CatLog: Cat No./ID: 74104, Germantown, MD, USA). In brief, cells were spun down in a tabletop centrifuge at 2000 rpm for 5 min and the supernatant removed. Cell pellet was washed with 1 mL cold PBS. A 250 mL aliquot of this cell suspension, representing 1-3 x 10^6 cells were used for RNA extraction according to the manufacturer's instructions. The corresponding cDNA clones were synthesized using SuperScript™ III First-Strand Synthesis system for reverse transcription (RT)-PCR (Invitrogen CatLog: 8080051) according to the manufacture's protocol using oligo (dT) and hexamer primers.

PCR Amplification of Variable Region of VH and VL Genes

The variable region sequences of HC and LC genes were amplified using cDNA clones derived from mouse hybridoma using the listed primers.[60] In brief, the VH and VL sense and antisense primers were used in 15:4 and 13:3 ratio, respectively. PCR reactions were performed in a volume

of 50 µl with 2 µl cDNA utilizing premixed Invitrogen PCR kits. The PCR reactions were carried out for 30 cycles, utilizing an annealing temperature (Tm) of 58 ±2 °C. The size of PCR products was verified by agarose gel electrophoresis.

Construction of Plasmids for Periplasmic Expression of Antibodies

The mouse antibody genes were synthesized, and then sub-cloned to an IPTG-inducible pET-based vector (pAB- cMyc-His tag) using *BssHII* and *NheI* restriction enzymes in such a way to append, in translational frame, Carboxy-terminal tags: a cMyc tag for detection; and a 6xHis-tag for purification via metal (nickel) affinity chromatography. The plasmid vector includes a PelB leader sequence under the control of a T7 promoter for expression in the periplasm of the *E. coli* expression host strain. The equivalent plasmid clone was designated as the mouse gene scFv 3A8. The DNA sequences of the plasmid was confirmed by sequencing.

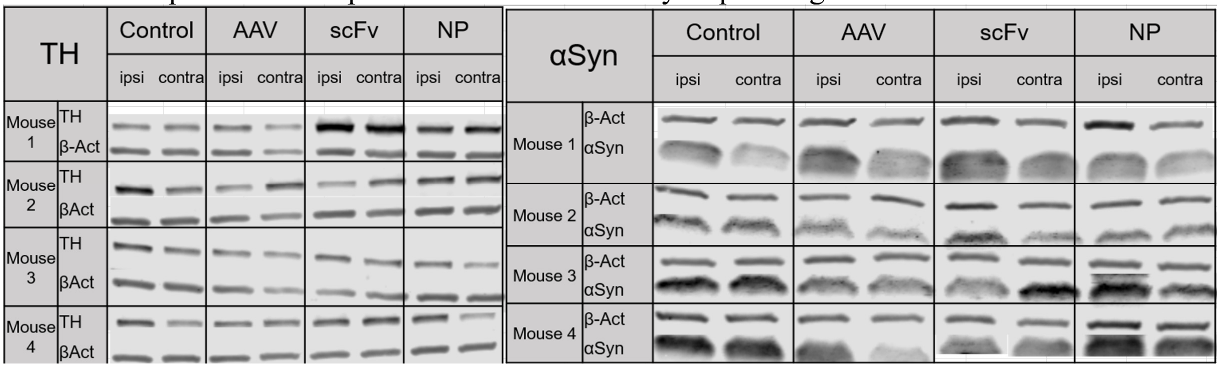

Supplementary Figure S1. Western blot images taken of the ipsilateral (ipsi) and contralateral (contra) SN in mice 12 weeks post AAV injection and compared to PBS-injected control. Images were used for densitometric analysis in ImageJ.

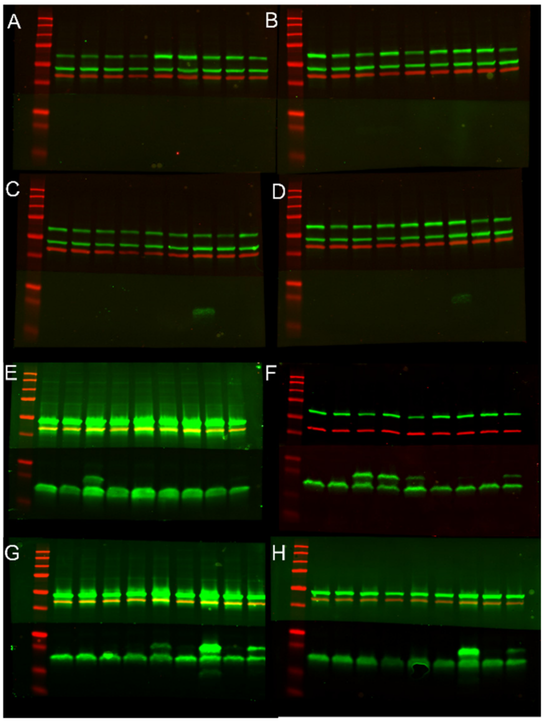

Supplementary Figure S2. Western blot original membranes. A-D) lower green band: TH. Red band: B-actin. E-H) lower green band: aSyn. Red band: B-actin. Green and red bands separated by color to remove interference; channels combined here for simplicity. Where applicable, double bands represented by aSyn were incorporated in Imagej integrated density calculations. Column descriptors: column 1 – ladder. A-H) column 2, 3 – control ipsi, contra. B-D, F-H) column 4,5 – AAV ipsi, contra; column 6,7 scFv ipsi, contra; column 8,9 NP ipsi, contra. A) column 4,5 – AAV ipsi, contra; column 6,7 NP ipsi, contra; column 8,9 scFv ipsi, contra. E) column 4,5 – NP ipsi, contra; column 6,7 AAV ipsi, contra; column 8,9 scFv ipsi, contra.

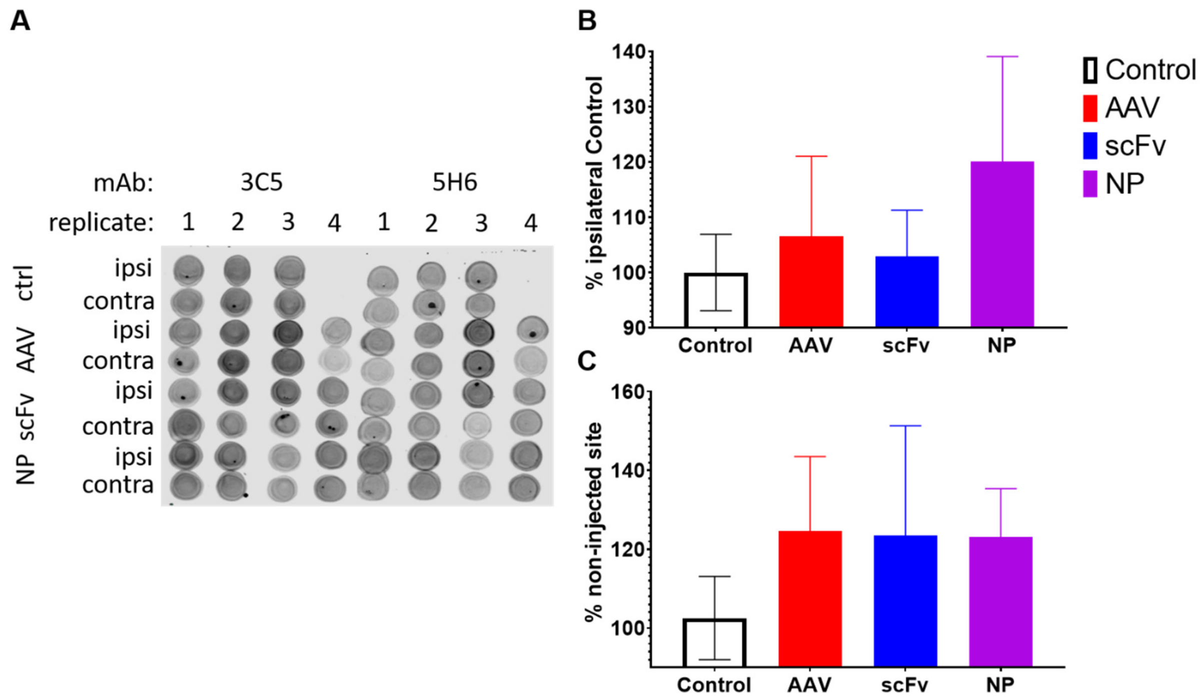

Supplementary Figure S3. A) Densitometric analysis of relative αSyn expression levels in the SN expressed as % ipsilateral/contralateral control B) densitometric analysis of relative TH expression levels in the SN expressed as % ipsilateral/contralateral control C) DA concentration in ng/mg in the STR, evaluated by HPLC, expressed as % ipsilateral/contralateral control D) DOPAC concentration in ng/mg in the STR, evaluated by HPLC, expressed as % ipsilateral/contralateral control. E) Representative dot blot images of samples taken from SN and evaluated for αSyn_{agg} expression by dot immunoblotting. Dot immunoblot of αSyn_{agg} corroborates increased αSyn expression as determined by western blot analysis. F) densitometric analysis showing % ipsilateral/contralateral control values of αSyn_{agg} for control, AAV, scFv and NP, respectively. n=3 mice for control, n=4 mice for AAV, scFv, and NP.
